# Platelet-12 lipoxygenase targeting via a newly synthesized curcumin derivative radiolabeled with technetium-99m

**DOI:** 10.1186/s13065-016-0220-x

**Published:** 2016-11-28

**Authors:** Reem Ibrahim Al-Wabli, Tamer Mostafa Mohamed Hafez Sakr, Mohammed Abdou Khedr, Adly Abdallah Selim, Mohamed Abd El-Motaleb Abd El-Rahman, Wafaa Abdou Zaghary

**Affiliations:** 1 Department of Pharmaceutical Chemistry, College of Pharmacy, King Saud University, Riyadh, 11451 Saudi Arabia; 2 Radioactive Isotopes and Generator Department, Hot Labs Center, Egyptian Atomic Energy Authority, P.O. Box 13759, Cairo, Egypt; 3 Department of Pharmaceutical Chemistry, Faculty of Pharmacy, Helwan University, Ein Helwan, Cairo, 11795 Egypt; 4 Labeled Compounds Department, Hot Labs Center, Egyptian Atomic Energy Authority, P.O. Box 13759, Cairo, Egypt

**Keywords:** Platelet-12 lipoxygenase, Curcumin, Technetium-99m, Cancer imaging, Enzyme targeting, Docking

## Abstract

**Background:**

One of the most popular techniques for cancer detection is the nuclear medicine technique. The present research focuses on Platelet-12-lipoxygenase (P-12-LOX) as a promising target for treating and radio-imaging tumor tissues. Curcumin was reported to inhibit this enzyme via binding to its active site.

**Results:**

A novel curcumin derivative was successfully synthesized and characterized with yield of 74%. It was radiolabeled with the diagnostic radioisotope technetium-99m with 84% radiochemical yield and in vitro stability up to 6 h. The biodistribution studies in tumor bearing mice confirmed the high affinity predicted by the docking results with a free binding energy value of (ΔG −50.10 kcal/mol) and affinity (13.64 pki) showing high accumulation in solid tumor with target/non-target ratio >6.

**Conclusion:**

The newly synthesized curcumin derivative, as a result of a computational study on platelet-12 lipoxygenase, showed its excellent free binding energy (∆G −50.10 kcal/mol) and high affinity (13.64 pKi). It could be an excellent radio-imaging agent that targeting tumor cells via targeting of P-12-LOX.

**Graphical abstract:**
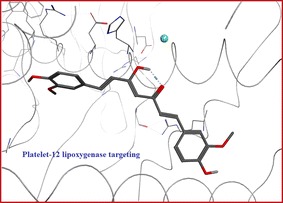
This novel curcumin derivative was successfully synthesized and radiolabeled with technetium-99m and biologically evaluated in tumor bearing mice that showed high accumulation in solid tumor with target/non-target ratio >6 confirming the affinity predicted by the docking results. Predicted binding mode of **a** new curcumin derivative in complex with 12-LOX active site. **b** Curcumin itself in the 12-LOX active site biological distribution of ^99m^Tc-curcumin derivative complex in solid tumor bearing Albino mice

## Background

Cancer is the main cause of mortality worldwide and the number of cancer patients is increasing at an alarming rate. Early detection of cancer greatly increases the chances of saving the patient’s life [1–3]. One of the most popular techniques for cancer detection is the nuclear medicine technique that is a breakthrough in the cancer imaging procedures [4, 5]. Technetium-99m is the most popular gamma emitting radionuclides used in nuclear medicine due to its perfect characteristics (6.02 h half-life and 140 keV γ-ray energy) and to its low cost and good availability [3–7]. In order to develop a successful radioactive tracer for cancer targeting, a selective organic compound that can differentiate between tumor and normal cells is extensively needed [8]. Curcumin is a natural product isolated from the rhizomes of the Indian *Curcuma longa* plant [9]. The Indian culture used curcumin as a food-flavoring agent, coloring agent and also in medicine as antiseptic, analgesic and antimalarial agent [10]. Researches proved that curcumin possesses other diverse of biological activities including antiviral [11], antibacterial [12], antifungal [13], anti-inflammatory [14], and antioxidant activities [15]. Recently, curcumin has drawn much attention due to its powerful anti-proliferative effect and anticancer activity in multiple cancers including ovarian, pancreatic, breast, melanoma, neck, colon, prostate, and head cancers [16–20]. The anticancer effect is manifested through the induction of apoptosis, growth arrest, and inhibition of the tubulin polymerization [21–23]. Furthermore, studies have shown that curcumin appeared as cytotoxic to cancer cells and cytoprotective to normal cells indicating that curcumin could be used as a selective safe radiotracer [24]. Specific enzyme targeting that is overexpressed in cancer, by using selective radiolabeled inhibitor of this enzyme, could be a great approach for treatment, imaging and diagnosis of cancers. The targeting process is a highly selective step that can be achieved by the computational approach [25]. The high over-expression of the Platelet-12 Lipoxygenase (P-12-LOX) was reported in different cancer tissues [26, 27]. Inhibition of such enzyme is considered to be a promising target for cancer treatment. To date few P-12-LOX inhibitors are known. Curcumin was reported to inhibit P-12-LOX via binding to its active site [28, 29]. The development of a novel curcumin derivative, which possesses higher free binding energy and good affinity to P-12-LOX, was one of the main objectives of this work. This is to select the highly predicted selective inhibitor of P-12-LOX to be synthesized then radiolabeled with technetium-99m followed by its in vivo evaluation as a novel target agent to P-12-LOX receptor in cancer cells.

## Experimental

### Chemicals

Curcumin and 2,4,6-trimethylbenzoyl chloride were purchased from Sigma-Aldrich, Steinheim, Germany. Analytical grade chemicals were directly used without further purification. All solutions were prepared using deionized water. Technetium-99m was eluted as ^99m^TcO_4_^−^ from ^99^Mo/^99m^ Tc generator, Elutec Brussels, Belgium.

### Instruments

Mettler FP 80 melting point apparatus was used to determine the melting points that were uncorrected. Ultrospec-2100 Pro UV visible spectrophotometer was used to record ultraviolet (UV) spectrum. Infrared (IR) spectra were recorded on FT/IR Shimadzu, Fourier transform, Infrared spectrometer/cm scale using KBr disc technique. ^1^H NMR and ^13^C NMR were carried out on Burker AC 500 MHz Spectrometer; chemical shifts are expressed in δ (ppm) downfield from TMS as an internal standard. Accela U-HPLC system coupled to a TSQ Quantum Access MAX triple stage quadrupole mass spectrometer carried out the LC–MS analysis (Thermo Scientific Corporation, USA) that was controlled with Xcalibur software version 2.2. Radioactivity measurements were done using Sodium Iodide (Tl) γ-ray scintillation counter (Scaler Ratemeter SR7, Nuclear Enterprises, Edinburgh, England). Ascending thin layer chromatography (TLC) run on pre-coated (0.25 mm) (GF 254) silica gel plates were used to follow up the reaction and the homogeneity of the compound. Routinely, used developing solvents system was C_6_H_6_:EtOAc:CHCl_3_ (5:1:5) (in ratio v/v). UV lamp at 254 nm was used to visualize the spots. Silica gel (60-230 mesh E. Merck) was used after heating at 110 °C for 1 h and was used for column chromatography separations. Silica gel 60 GF_254_ for TLC was used for coating 20 × 20 cm glass plates for preparative TLC.

### Animals

The biological distribution was evaluated in 20–25 g Albino mice.

### Software programs

Molecular Operating Environment (MOE) package license was purchased from Chemical Computing Group Inc, Sherbooke St, Montreal, QC, Canada [30].

### Molecular docking

All compounds were built and saved as MOE. Rigid receptor was used as a docking approach. Receptor and solvent were kept as a “receptor’’. Triangle matcher was used as a placement method with timeout of 300 s. Two rescoring were computed; rescoring 1 was selected as London dG while rescoring 2 was selected as affinity. Force field was used as a refinement. The best conformation for each compound was kept inside the docking pocket and the affinity (pKi) was computed. The free energy of binding ∆G (kcal/mol) for the proposed derivatives were also recorded.

### Synthesis of curcumin derivative (1,7-bis[((4′-(2″,4″,6″-trimethylbenzoyl)oxy)-3′-methoxyphenyl]-1,6-heptandiene-3,5-dione)

**Scheme 1:**
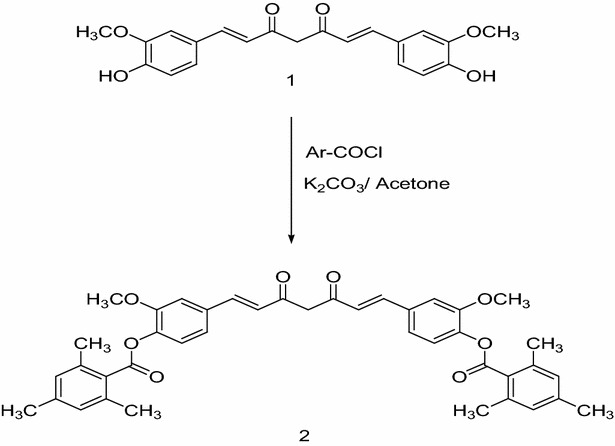
Chemical synthesis pathway

To an ice-cold curcumin solution **(1)** (3.68 g, 0.01 mol) in 50 ml dry acetone, 2 g of sodium carbonate was added with stirring for 15 min. The 2,4,6-trimethylbenzoyl chloride (0.025 mol) was added dropwise to the mixture over a period of 30 min. The reaction was then refluxed for 12 h [31]. The reaction mixture was filtered, evaporated then extracted using EtOAc (3 × 30 ml). The combined organic layers were dried using anhydrous MgSO_4_ and the solvent volume was reduced under reduced pressure to give a yellow precipitate, which was recrystallized from ethanol to give compound (**2)** (Scheme 1) with yield 74%, mp 210–212 °C, molecular formula C_41_H_40_O_8_ and M.Wt. 660.

### Preparation of ^99m^Tc-curcumin derivative complex

Curcumin (150 μg) was dissolved in 1 ml DMSO in 10 ml penicillin vials. Then about 10 mg of NaBH_4_ was added to each vial with pH adjustment in a range of 6–10 using of 0.1 N sodium hydroxide or 0.1 N Hydrochloric Acid. Followed by the addition of 100 μl of freshly eluted ^99m^TcO_4_^−^ (~200 MBq) to each vial. These reactions were performed for 15 min at room temperature. These procedures were repeated to evaluate the radiochemical yields with varying NaBH_4_ amounts (5–30 μg), varying curcumin amounts (50–300 μg), on different time scale (5–60 min).

### Radiochemical yield assay of ^99m^Tc-curcumin derivative complex

^99m^Tc-curcumin derivative complex radiochemical yield and in vitro stability were evaluated by using strips of ascending Whatman paper chromatography (PC). Two strips were used per experiment, on which two drops of the reaction product were placed on origin line at distance of 2 cm from the bottom.

For the determination of the ratio of free ^99m^TcO_4_^−^ radio-contaminant, acetone was used as a developing solvent for one PC strip, where free ^99m^TcO_4_^−^ R_f_ was 1 while ^99m^Tc-curcumin derivative complex and reduced hydrolyzed technetium colloid species R_f_ was zero.

Another strip was developed in C_2_H_5_OH:H_2_O:NH_4_OH mixture (2:5:1, v/v/v) to determine the ratio of the hydrolyzed ^99m^Tc radio-contaminant, where reduced hydrolyzed technetium colloid R_f_ is zero while free ^99m^TcO_4_^−^ and ^99m^Tc-curcumin derivative complex species R_f_ is 1.

At the end of the developing process, the strips were dried, cut into 1 cm pieces and counted using the sodium iodide (Tl) γ-ray scintillation counter. Each experiment was repeated three times.

The radiochemical yield percent of ^99m^Tc-curcumin derivative complex was calculated according to the following equation:

$$ {\%\text{Radio chemical yield}} = 100 - \left( {{{\% \text {Free}\; ^{99{\rm m}} \text {TcO}}}_{{4}}^{ - }  + {\text{\%colloid}}} \right) $$

### Biological distribution

The animal ethics committee guided this study was following the guidelines of the Egyptian Atomic Energy Authority. The biological distribution was evaluated in mice bearing solid tumor.

#### Solid tumor induction in mice

The solid tumor induction was done using Ehrlich ascites carcinoma (EAC) that was derived from a murine mammary carcinoma [32, 33]. The parent tumor line EAC had been derived from 7 days old donor female Swiss Albino mice and diluted with sterile physiological saline solution. To induce a solid tumor, about 0.2 ml solution was I.M. injected in the female Albino mice right thigh for 4–6 days [3, 5, 34].

#### Biodistribution assay of ^99m^Tc-curcumin derivative complex

The biodistribution study of ^99m^Tc-curcumin derivative complex was evaluated at time intervals of 5, 15, 30, 60, 120 and 180 min post injection (p. i) in solid tumor bearing Albino mice (n = 5 mice/time point). Mice were separated in groups and supplied with food and water. ^99m^Tc-curcumin derivative complex was I.V. injected in the mice tail vein.

Firstly, animals were anaesthetized using chloroform, then weighted and sacrificed at different time intervals. All body organs and tissues were separated, collected and washed with saline then weighted. Blood, bone and muscle samples were collected and weighted then were assumed to be 7, 10 and 40% of the total body weight, respectively [3, 35]. The organs radioactivities as well as the background were measured in a well type γ-counter NaI(Tl). In a population of five, the percent injected dose/gram organ or tissue (%ID/g) were calculated. Target (solid tumor) to non-target (normal muscle) ratio (%T/NT) was calculated from %ID/g for solid tumor and normal muscle.

### Statistical analysis

Graph Pad Prism version 6.0 software was used to do all the statistical analyses. Statistical analysis was conducted using one-way ANOVA followed by multiple Tukey–Kranes post hoc test at *ʋ* < 0.05 considered for statistical significance.

## Results and discussion

### Computational selection of the best curcumin derivative

**Fig. 1:**
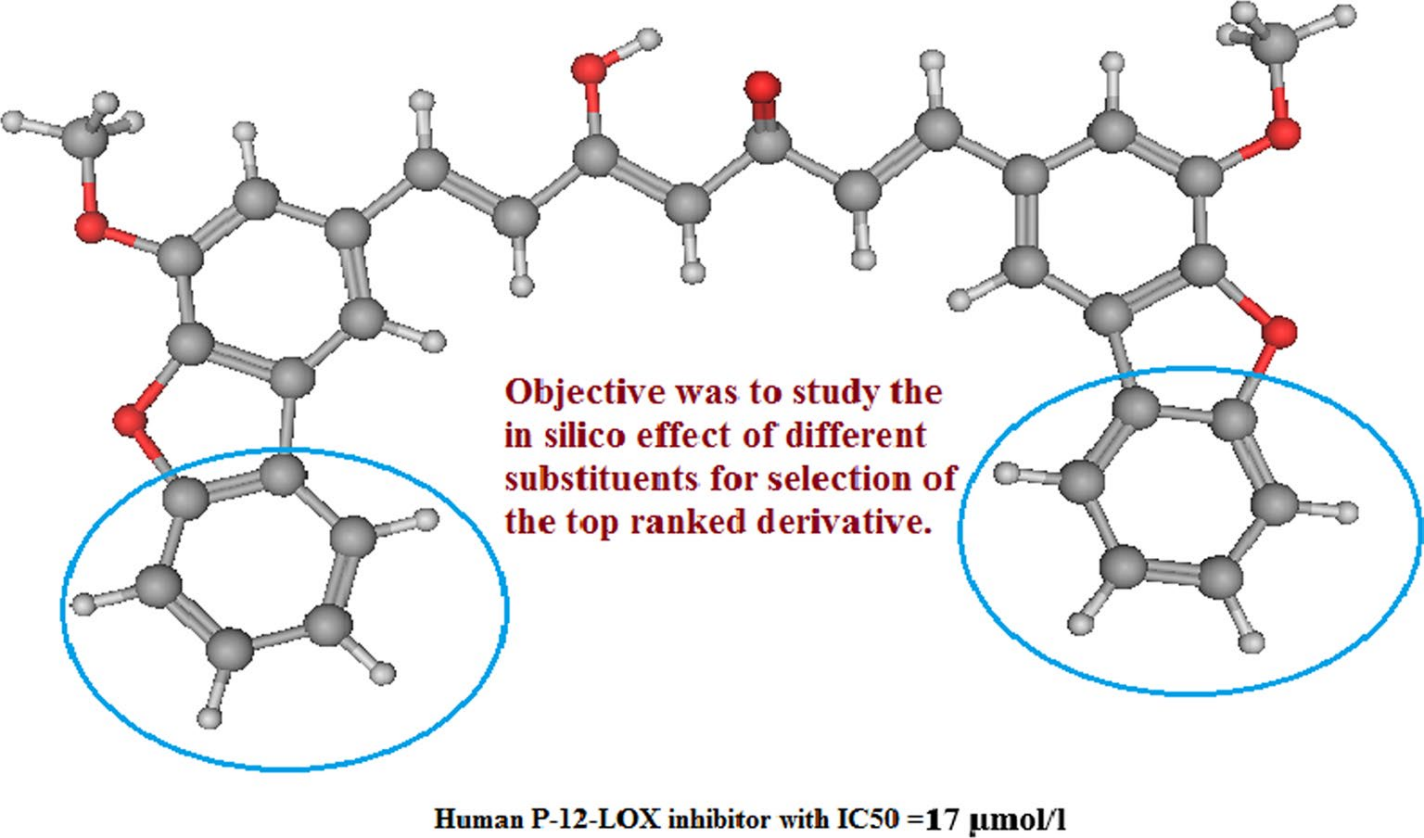
Structure of E26C, a human P-12-LOX inhibitor. It is a curcumin derivative with benzofuran ring

Selectivity plays a major role in drug targeting process. It can help in identifying the most suitable ligand (key) for a specific enzyme (lock). Computational approaches are widely used nowadays to compute the free energy of binding, affinity and other parameters like LogP that have an indication of good fitting and predictive high selectivity. P-12-LOX is overexpressed in many tumor tissues [36]. Arachidonic acid is metabolized by P-12-LOX to produce a hydroxyeicosatetraenoic acid that has been reported to be a main cause of cancer development [37, 38]. Thus, inhibition of P-12-LOX can decrease both cell proliferation and metastasis [39]. Curcumin was reported to inhibit P-12-LOX (66 µmol/l) [28, 29]. Also, a number of synthetic curcuminoids were reported to have a promising P-12-LOX inhibitory activity [40]. Compound E26C, a curcumin derivative with benzofuran moiety, had P-12-LOX inhibitory activity IC50 = 17 µmol/l and showed the best fitting distance 3.3 Å among all the reported curcuminoids (Fig. 1). The discovery of a curcumin derivative that can possess high affinity toward P-12-LOX and can be radiolabeled for tumor imaging was the main objective of this study. Radiolabeling of a highly selective P-12-LOX inhibitor will also ensure high accuracy of tumor cells imaging, as this enzyme is overexpressed in tumor tissues as mentioned before.

The study aimed to propose different curcumin derivatives that possess different substitution on the aromatic ring and that simulate the lead structure (Fig. 1).

**Fig. 2:**
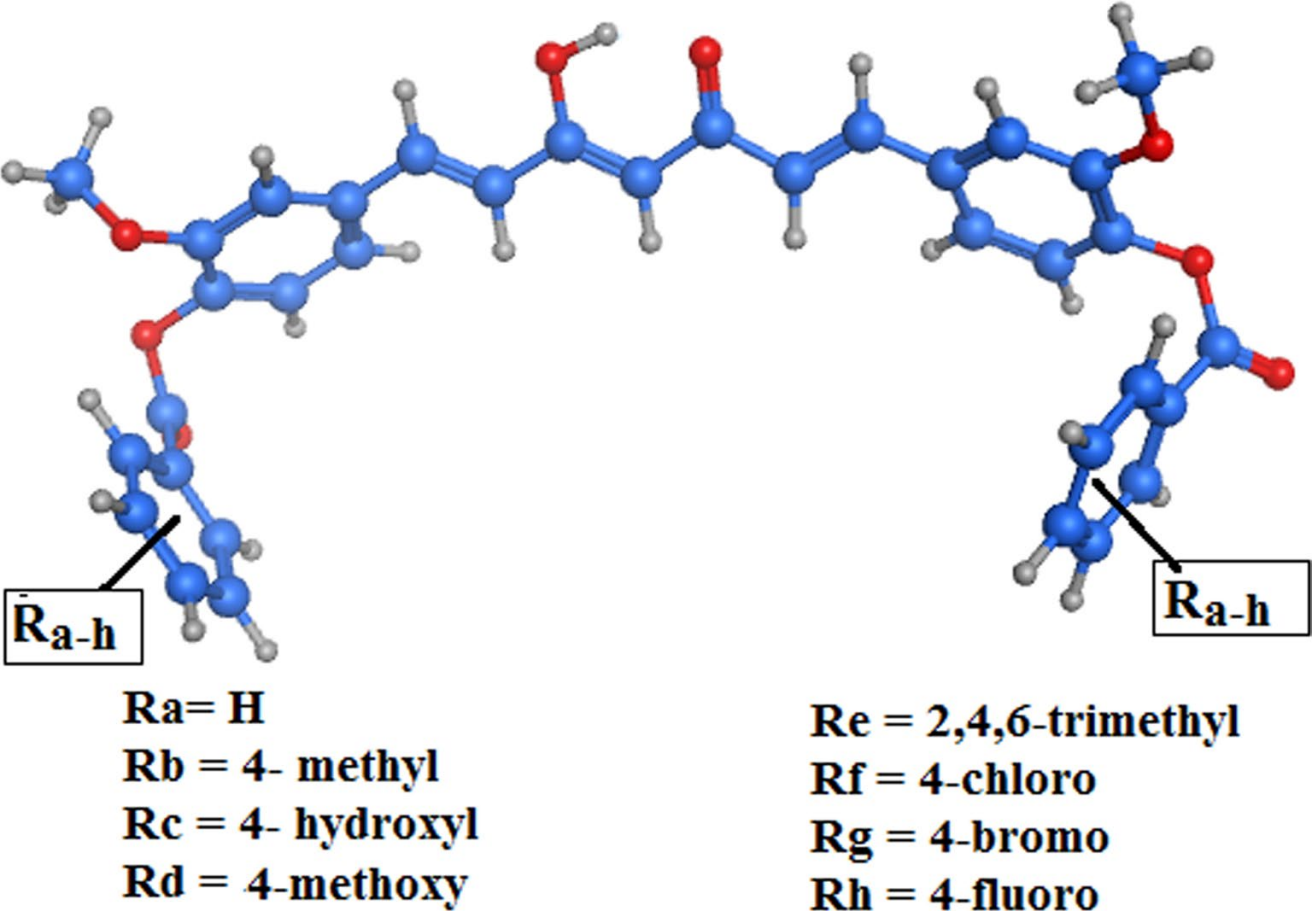
Proposed curcumin derivatives with substituted phenyl side chain

A number of curcumin derivatives with a substituted phenyl ring at the same position of the lead derivative were designed (Fig. 2). The computational affinity (pKi), cLogP and free binding energy (ΔG) were computed and compared to those of both curcumin itself and the reported benzofuran derivative (Table 1).

**Table 1:** Computed affinity (pKi), cLogP, and ∆G (kcal/mol) for the proposed derivatives

	Compound	Affinity (pKi)	cLogP	∆G kcal/mol
1	Phenyl	11.38	6.8	−30.54
2	4-Methyl phenyl	11.51	7.49	−40.65
3	4-Hydroxy phenyl	10.37	6.2	−43.40
4	4-Methoxy phenyl	11.50	6.8	−43.45
5	2,4,6-Trimethyl phenyl	*13.64*	*7.7*	−*50.10*
6	4-Chloro phenyl	10.55	8.1	−41.75
7	4-Bromo phenyl	10.72	8.4	−41.95
8	4-Fluoro phenyl	10.24	7.1	−42.15
9	Curcumin	8.66	3.85	−30.85
10	Curcumin derivative (E26C)	*10.40*	*7.9*	−*51.65*

It was clear that E26C had more inhibitory activity than curcumin, it showed higher affinity (pKi) 10.40 and higher cLogP (7.9) as well. Besides, its free binding energy was very less and favorable (−51.65 kcal/mol) than that of curcumin (−30.85 kcal/mol).

As a result, from all the proposed structures, we were looking for the one with higher affinity, less free binding energy and higher cLogP than curcumin and comparing it with E26C as well. The 2,4,6-trimethyl phenyl derivative showed almost the same cLogP (7.7) to that of E26C and with high affinity (13.64) that was higher than both of E26C and curcumin. The 2,4,6-trimethyl phenyl derivative also showed less free binding energy (−50.10 kcal/mol) when compared to other proposed derivatives.

**Fig. 3:**
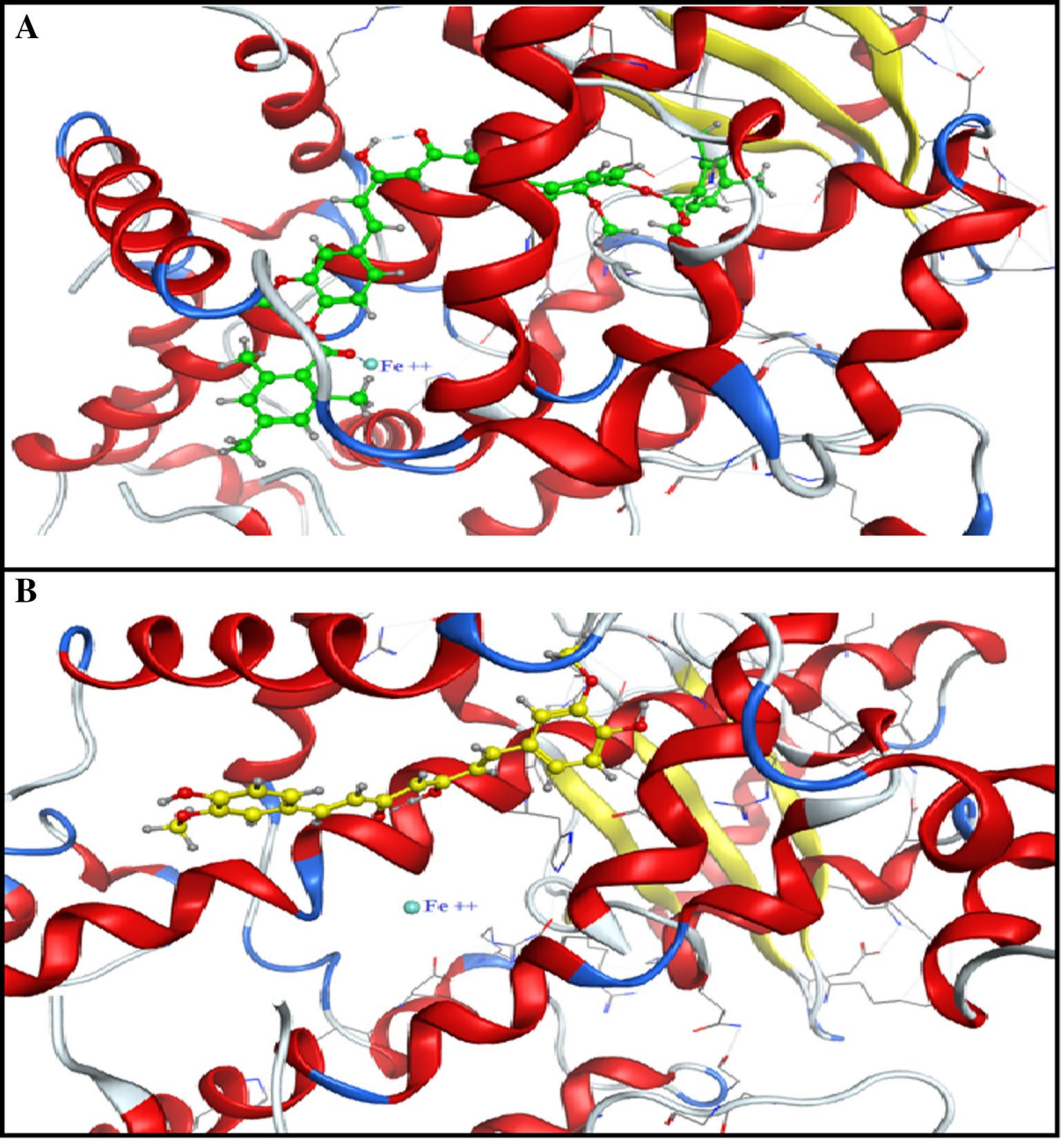
Predicted binding mode of **A** new curcumin derivative in complex with 12-LOX active site. **B** Curcumin itself in the 12-LOX active site

The new curcumin derivative showed better free binding energy that was reflected upon the total potential energy, which was lower toward the most stable state in case of the new derivative when compared to that of curcumin complex. In addition, it had a higher affinity (13.64). The binding of the new compound showed good coordination with the iron metal in the active site that was not achieved by the curcumin itself (Fig. 3).

In accordance, the curcumin derivative with (1,7-Bis[((4′-(2″,4,6″-trimethylbenzoyl)oxy)-3′-methoxyphenyl]-1,6-heptandiene-3,5-dione) was selected to be synthesized and radiolabeled with techniethium-99m.

**Fig. 4:**
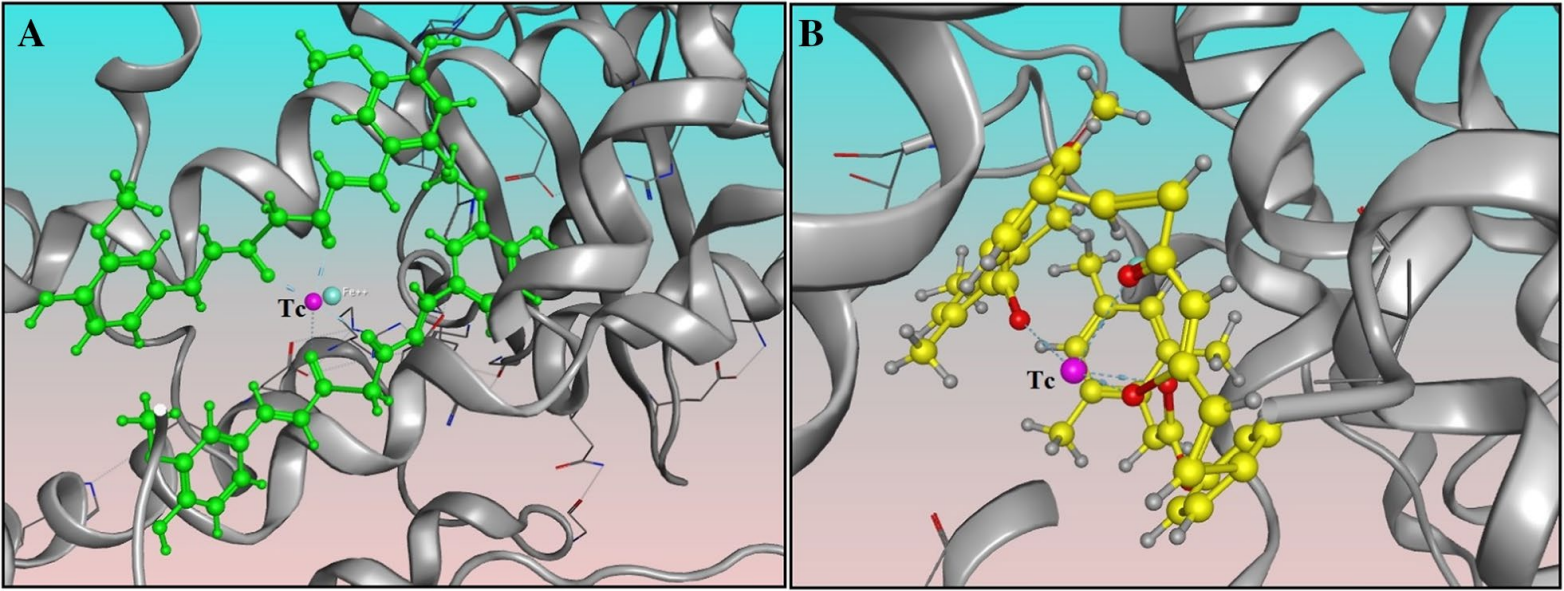
**A** Curcumin-Tc complex. **B** Selected derivative-Tc complex

The proposed chemical complex that may be formed between technetium 99m and the top ranked selected curcumin derivative compound with (1,7-Bis[((4′-(2″,4″,6″-trimethylbenzoyl)oxy)-3′-methoxyphenyl]-1,6-heptandiene-3,5-dione) was compared to curcumin-technetium-99m complex. It was proposed that two molecules of curcumin were complexed with one technetium-99m. While, (1,7-Bis[((4′-(2″,4″,6″-trimethylbenzoyl)oxy)-3′-methoxyphenyl]-1,6-heptandiene-3,5-dione) was complexed with by 1:1 ratio in which the technetium-99m formed a complex with the two carbonyl of the benzoyl moieties in addition to that at the 3,5-dione. In the first case the two curcumin molecules formed a long and wide complex while the 2,4,6-trimethyl benzoyl derivative showed a conformation that illustrated a well-fitting. When both complexes were docked, curcumin showed an affinity pki of (33.54) that was lower than that of the selected curcumin derivative that achieved an affinity pki of (45.20) to prove that it has more stability than that of curcumin in enzyme binding in the technetium-99m complex form (Fig. 4).

### Chemical synthesis of curcumin derivative

The chemical synthesis was achieved in one-step reaction through reacting 2,4,6-trimethylbenzoyl chloride with curcumin according to the method mentioned previously.

IR (KBr) ʋ (cm^−1^): 3008 (CH-Ar), 2922, 2854 (CH-aliphatic), 1741 (C=O of ester), 1631 (C=O) 1600 (C=C aromatic), and 1122 (C–O). ^1^H NMR (CDCl_3_) δ ppm (500 MHz): 2.35–2.49 (s, 18H, 6 × CH_3_), 3.94 (s, 6H, 2 × OCH_3_), 5.91 (s, 1H, H-4), 6.62 (d, 2H, H-2 & H-6, *J* = 15.5 Hz), 6.96 (s, 4H, H-3″ & H-5″), 7.21–7.29 (m, 6H, Ar–H) and 7.68 (d, 2H, H-1 & H-7, *J* = 16 Hz). ^13^C NMR (DMSO-*d*_6_) δ ppm (125 MHz): 20.0 (4 × CH_3_), 21.2 (2 × CH_3_), 55.8 (2 × OCH_3_), 101.9 (C-4), 111.6 (2 × C-2′), 121.2 (2 × C-6′), 123.3 (2 × C-5′), 124.4 (2 × C-1′), 128.7 (C-2, C-6, 2 × C-3″ & 2 × C-5″), 129.6 (2 × C-1″), 134.1 (2 × C-4″), 136.0 (2 × C-2″ & 2 × C-6″), 140.0 (C-1 & C-7), 141.3 (2 × C-3′), 151.7 (2 × C-4′), 167.6 (2 × C=O of ester) and 183.1 (C-3 & C-5). MS: *m/z* (%) for C_41_H_40_O_8_: 661 [M^+^ + 1] (26%).

### Factors affecting on radiochemical yield of ^99m^Tc-curcumin derivative

#### Effect of reducing agent (NaBH_4_) amount

**Fig. 5:**
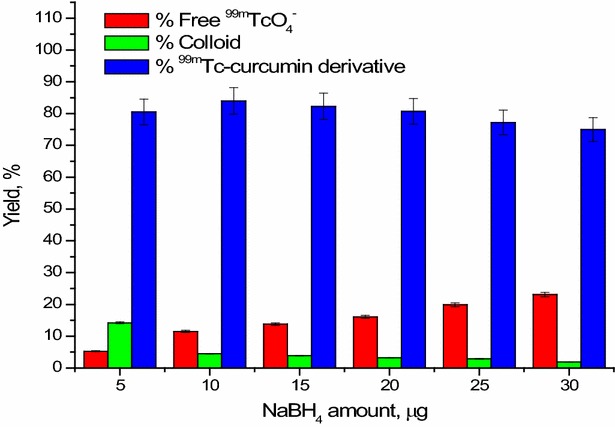
Effect of NaBH_4_ amount on the radiochemical yield of ^ 99m^Tc-curcumin derivative complex

The effect of NaBH_4_ on the % radiochemical yield of ^99m^Tc-curcumin derivative complex was shown in (Fig. 5). At 5 mg NaBH_4_, the %^99m^TcO_4_^−^ was equal to 5.3 ± 0.8% that may be due to insufficient amount of NaBH_4_ to complete the reduction of ^99m^TcO_4_^−^ to form ^99m^Tc-complex. Increasing the amount of NaBH_4_ to 10 mg, the maximum radiochemical yield (84 ± 1.4%) was revealed. Increasing the NaBH_4_ greater than 10 mg the radiochemical yield was decreased to 75 ± 1.11% at 30 μg NaBH_4_.

#### Effect of pH

**Fig. 6:**
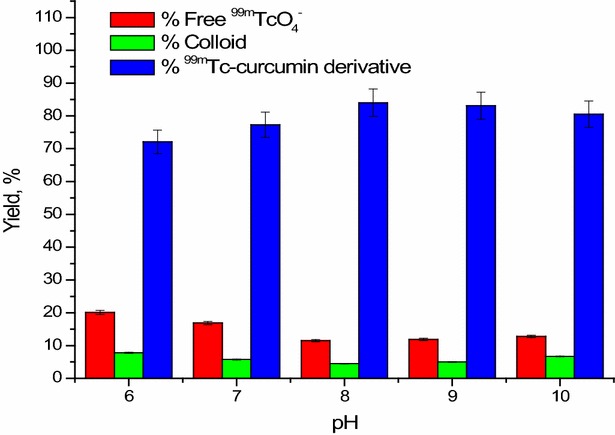
Effect of pH on the radiochemical yield of ^99m^Tc-curcumin derivative complex

As shown in Fig. 6, the radiochemical yield of ^99m^Tc-curcumin derivative complex was affected by pH change. At pH 6, the radiochemical yield was relatively low (72.1 ± 1.2%). While, the maximum radiochemical yield (84 ± 1.4%) was observed at pH 8, where the curcumin combined all the reduced technetium. When the pH increased above 8, the percent radiochemical yield was slightly decreased.

#### Effect of curcumin derivative amount

**Fig. 7:**
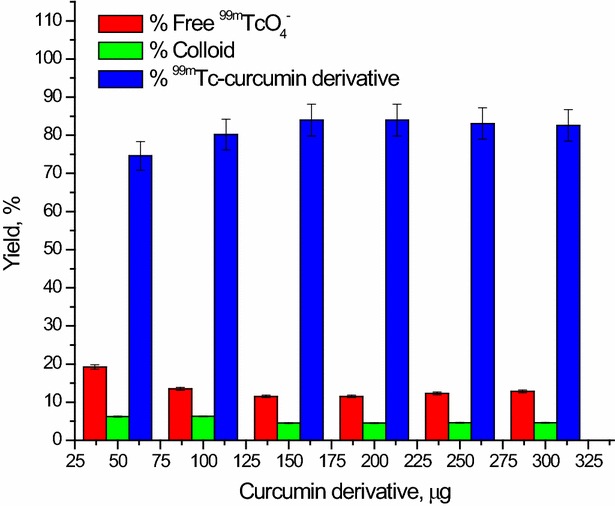
Correlation between curcumin derivative amount and the radiochemical yield of ^99m^Tc-curcumin derivative complex

The correlation between the radiochemical yield and the amount of curcumin derivative is shown in Fig. 7. The maximum radiochemical yield of ^99m^Tc-curcumin derivative complex (84 ± 1.4%) was obtained at 150 µg curcumin. At low curcumin amount (50 µg), the radiochemical yield was low (74.6 ± 1.3%), where the amount of curcumin was insufficient for forming complex with the reduced technetium. The radiochemical yield was increased by increasing the curcumin amount where a maximum radiochemical yield of 84 ± 1.4% was obtained at 150 µg curcumin.

#### Effect of reaction time

**Fig. 8:**
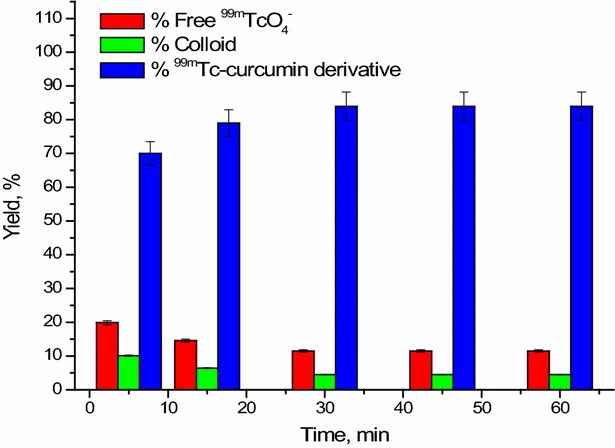
Effect of reaction time on the radiochemical yield of ^99m^Tc-curcumin derivative complex

The formation of ^99m^Tc-curcumin derivative complex was started relatively slowly as the radiochemical yield was 70 ± 1.1% at 5 min reaction time as shown in Fig. 8. The maximum radiochemical yield of ^99m^Tc-curcumin derivative complex was obtained at 30 min. Also, it remained constant up to 1 h.

### In vitro stability study

The radiochemical yield of the ^99m^Tc-curcumin derivative complex showed stability for up to 6 h that confirmed its suitability for use during this time period.

### Biology study

**Fig. 9:**
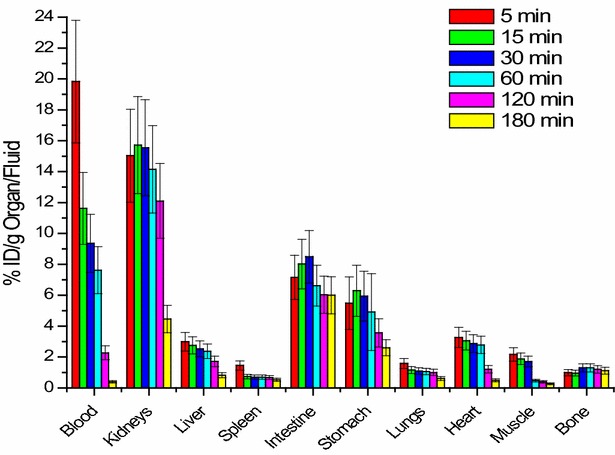
Biological distribution of ^99m^Tc-curcumin derivative complex in solid tumor bearing Albino mice

The distribution of ^99m^Tc-curcumin derivative complex was studied in solid tumor-bearing mice (%ID/g) at 5, 15, 30, 60, 120 and 180 min post injection. The accumulation of the %ID/g of ^99m^Tc-curcumin derivative in different body organs and fluids is illustrated in Fig. 9. It was clear that ^99m^Tc-curcumin derivative didn’t accumulate in a specific body organ and was mainly excreted via both the urinary tract and hepatobiliary pathways. The kidneys showed 15.72 ± 3.1% ID/g at 15 min and intestine showed 8.49 ± 1.8% ID/g at 30 min.

**Fig. 10:**
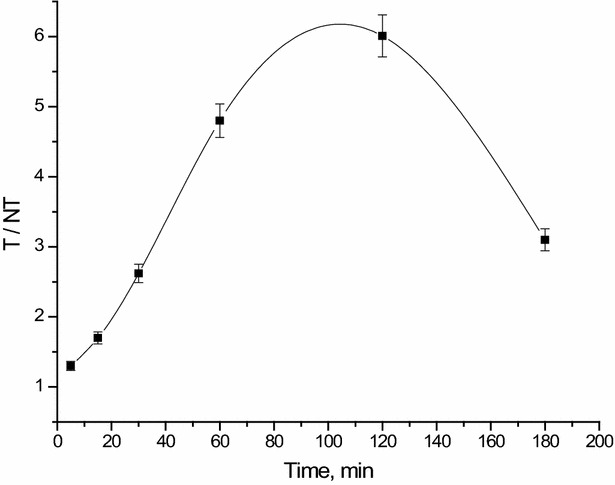
T/NT of ^99m^Tc-curcumin derivative complex in solid tumor bearing Albino mice

The tumor tissue (mouse right leg muscle)/normal tissue (mouse left leg muscle) ratio represents the key factor in the evaluation of the selectivity and sensitivity of ^99m^Tc-curcumin derivative complex to solid tumor. As shown from Fig. 10, the T/NT ratio of ^99m^Tc-curcumin derivative complex in solid tumor-bearing mice was ~1.7 at 15 min post injection and increases to its highest value of ~6.01 at 120 min post injection (p.i.) that clearly prove its high selectivity for the tumor cells.

This high preclinical T/NT ratio presents ^99m^Tc-curcumin derivative complex as a non-invasive probe for solid tumor imaging when compared with many other agents such as: ^99m^Tc-meropenem (3.5 at 1 h p.i.) [7], ^99m^Tc-sunitinib (3 at 1 h p.i.) [3], ^99m^Tc-PyDA (3 at 1 h p.i.) [5], Radioiodinated anastrozole (4.7 ± 0.06 at 2 h p.i.) [41], radioiodinated epirubicin (5.2 ± 0.09 at 1 h p.i.) [42], ^99m^Tc-BnAO-NI (2.59, 2 h) [43], [^99m^Tc(CO)_3_(IDA–PEG3–CB)]^−^ (3.45, 3 h) [44], ^99m^Tc(CO)_3_-labeled chlorambucil analog (3.2 at 3 h p.i) [45], ^99m^Tc-nitride-pyrazolo [1,5-*a*] pyrimidine (2.2 at 1 h p.i.) [46], ^99m^Tc-DETA (2.47 at 4 h p.i.) [32], ^99m^Tc-TETA (2.45 at 4 h p.i.) [32], ^99m^Tc-TEPA (2.91 at 4 h p.i.) [32], ^99m^Tc-citro-folate (4.3 at 4 h p.i.) [47] and ^99m^Tc-gemcitabine (4.9 at 2 h p.i.) [48]. All of these present ^99m^Tc-curcumin derivative complex as a promising solid tumor imaging agent.

## Conclusion

A promising curcumin derivative with high affinity 13.64 (pKi) and excellent free binding energy (−50.10 kcal/mol) was selected for chemical synthesis and radiolabeling to target P-12-LOX. It showed a high radiochemical yield of 84% and in vitro stability up to 6 h. Its high accumulation in solid tumor with target/non-target ratio >6 indicated that it could be an excellent radio-imaging agent that targets tumor cells via selectivity of P-12-LOX.
